# Supplementary material for: Fluorescent Auxin Analogs Report Two Auxin Binding Sites with Different Subcellular Distribution and Affinities: A Cue for Non-Transcriptional Auxin Signaling
Source: Int J Mol Sci. 2022 Aug 2;23(15):8593. doi: 10.3390/ijms23158593 (PMC9369420; doi:10.3390/ijms23158593)
Supplement: Supplementary file 1 [file ijms-23-08593-s001.zip › ijms-1818833-supplementary.pdf]

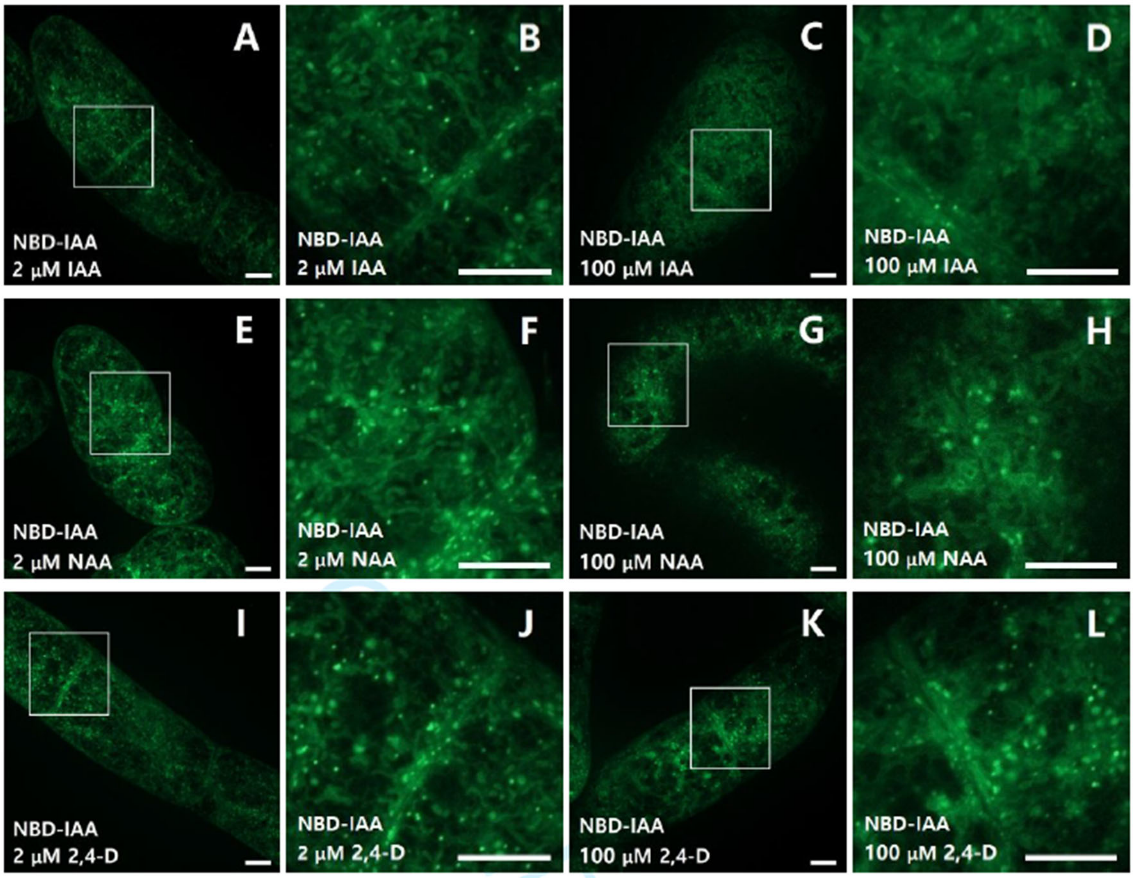

Supplementary Figure S1. Effects of auxin (IAA, NAA, and 2,4-D) on NBD-IAA distribution in WT BY-2 cells. The wild type BY-2 cells were incubated with 2 μM (**A** and **B**) or 100 μM (**C** and **D**) IAA and 2 μM NBD-IAA together for 20 min. The cells were co-treated with 2 μM (**E** and **F**) or 100 μM (**G** and **H**) NAA and 2 μM NBD-IAA for 20 min. The cells were treated with 2 μM (**I** and **J**) or 100 μM (**K** and **L**) 2,4-D, together with 2 μM NBD-IAA for 20 min. Scale bar represents 10 μm.
